# Supplementary material for: A Patient-Centered Conceptual Model of AYA Cancer Survivorship Care Informed by a Qualitative Interview Study
Source: Cancers (Basel). 2024 Sep 4;16(17):3073. doi: 10.3390/cancers16173073 (PMC11394144; doi:10.3390/cancers16173073)
Supplement: Supplementary file 1 [file cancers-16-03073-s001.zip › cancers-3144600-supplementary.pdf]

Supplement S1: Interview Guide - AYA Survivorship Experience

Before we begin, I'd like to ask about the word "survivor". Sometimes people use the word **"survivor"** to describe someone who has had cancer – do you identify with this term?

[No:] How do you see yourself/what word would you use instead? [Yes]: what does survivorship mean to you?

Could you share with me your experience of having cancer, going through treatment, and being a survivor. What has that been like for you?

	**Post-cancer (now)**<br>How has cancer impacted/contributed to:<br>*(use for question stems below)*	**Precancer**<br>Was your experience before cancer:	**Post-cancer (future)**<br>What do you anticipate being…
**Individual/ Personal Health**	□ How would you describe your health now, generally?<br>□ - **other conditions (comorbidities).** Present pre-cancer?<br>□ - need/ability to manage treatment **side effects/symptoms?**<br>□ - need/ability to manage **late effects of your treatment?**<br>□ - **family building goals, if any** *(probe for family at time of dx)*<br>□ **sexual health** (especially for male-identifying participants)<br>□ **sexual expression** (gender identity/sexuality)<br>□ **sex life** (comfort, pain, performance)<br>□ support received to help with above challenges?<br>□ Other things I haven't asked about?<br>□ How has COVID affected your post-cancer life?	□ Was your experience different during treatment? Could you share more?<br>□ same?	□ different in the future?<br>□ same?
**Individual/ Personal Beliefs/Self-narrative/goal s**	□ - main life goals (i.e. education, career, family)<br>□ - challenges you've experienced achieving your goals?<br>□ - your ability to cope with challenges in your life?<br>□ Do you anticipate being able to achieve those goals?<br>□ What might you need support with to achieve your goals?	□ different from now?<br>□ same?	□ different in the future?<br>□ same?
**Individual/ Personal Emotions/ spiritual**	□ - **hopes**<br>□ - **fears/ anxieties** (*write:)*____________________<br>□ - sense of control (*write:)*____________________<br>□ **- emotions (mental health/depression)** (*write:)*__________<br>□ - sense of spirituality and/or religious faith (*write:)*________<br>□ Have you experienced past events (i.e. illness of a loved one) that have impacted your cancer experience?<br>□ Have you received support for (*challenge(s) shared above*)?	□ different from now?<br>□ same?	□ different in the future?<br>□ Same?
**Interpersonal Altered social relationships**	□ - relationships with the people you consider family?<br>□ - relationships with friends?<br>□ Can you talk about connections w other AYAs who've had cancer? Support groups you've been part of?	□ different from now?<br>□ same?	□ different in the future?<br>□ same?
**System/Role Logistics/role**	□ Do you have challenges related to childcare?<br>□ How have these challenges impacted you (ability to attend appointments, participate in child's life?)<br>□ - your access to transportation (to work/appointments)?<br>□ Most challenging? Travel time? Distance to appt? Expense?	□ different from now?<br>□ same?	□ different in the future?<br>□ same?
**System Employment/ Career**	□ - employment or job (probe for opportunities/disruptions)<br>□ Can you talk the economic implications/**financial burden of your treatment?**<br>□ Medical debt/Out of pocket costs/worried about bills<br>□ Tradeoffs/decisions made to afford medical care<br>□ Has **insurance coverage** been a challenge for you?	□ different from now?<br>□ same?	□ different?<br>□ same?

	□ Denied coverage?<br>□ Disruptions in coverage? Why? Impact of ACA?		
**System Medical Care**	□ What does **good cancer care** for an AYA patient look like, from your perspective?<br>□ Did you feel that you were treated with dignity and respect during your care? OR: or did you experience any unjust/unfair treatment<br>□ Can you talk about the **diversity of your medical team?** Were there people on your medical team that you felt understood your particular concerns and perspective?<br>□ ability to **get the medical care you need when you need it?**<br>□ Timely/Delays? Easy to access? Barriers/Challenges?<br>□ Can you talk about your ability to **communicate with your doctor?**<br>□ Was there anything that you wished your cancer care providers communicated to you that they didn't?<br>□ Were there things you wish were communicated differently?<br>□ How well did your cancer care providers worked with each other to help **coordinate your care**?<br>□ What worked well? What were the challenges?<br>□ Looking back, are there things you wish you knew previously about any aspect of cancer care?	□ different from now?<br>□ different during treatment?<br>□ same?	□ different in the future?<br>□ same?
**Future Planning**	□ How do you think about/plan for your future health?<br>□ How are you planning for your future in general?	□ different?<br>□ same?	n/a

If not covered above, ask these additional probes:

- What do you wish your doctors knew about what AYAs need from their care team?
- From your experience, what do you think is needed to improve cancer care for others in the future?
- What did I not ask you about, that you think is important about your experience as a young adult who has had cancer?
